# Partial Least Square Discriminant Analysis Discovered a Dietary Pattern Inversely Associated with Nasopharyngeal Carcinoma Risk

**DOI:** 10.1371/journal.pone.0155892

**Published:** 2016-06-01

**Authors:** Yen-Li Lo, Wen-Harn Pan, Wan-Lun Hsu, Yin-Chu Chien, Jen-Yang Chen, Mow-Ming Hsu, Pei-Jen Lou, I-How Chen, Allan Hildesheim, Chien-Jen Chen

**Affiliations:** 1 Institute of Biomedical Sciences, Academia Sinica, Taipei, Taiwan; 2 Genomics Research Center, Academia Sinica, Taipei, Taiwan; 3 National Institute of Cancer Research, National Health Research Institutes, Chunan, Taiwan; 4 Graduate Institute of Microbiology, College of Medicine, National Taiwan University, Taipei, Taiwan; 5 Department of Otolaryngology, National Taiwan University Hospital and National Taiwan University College of Medicine, Taipei, Taiwan; 6 Department of Otorhinolaryngology, Head and Neck Surgery, Chang Gung Memorial Hospital and Chang Gung University, Taoyuan, Taiwan; 7 Division of Cancer Epidemiology and Genetics, National Cancer Institute, NIH, DHHS, Bethesda, Maryland, United States of America; 8 Graduate Institute of Epidemiology and Preventive Medicine, College of Public Health, National Taiwan University, Taipei, Taiwan; Tufts University, UNITED STATES

## Abstract

Evidence on the association between dietary component, dietary pattern and nasopharyngeal carcinoma (NPC) is scarce. A major challenge is the high degree of correlation among dietary constituents. We aimed to identify dietary pattern associated with NPC and to illustrate the dose-response relationship between the identified dietary pattern scores and the risk of NPC. Taking advantage of a matched NPC case–control study, data from a total of 319 incident cases and 319 matched controls were analyzed. Dietary pattern was derived employing partial least square discriminant analysis (PLS-DA) performed on energy-adjusted food frequencies derived from a 66-item food-frequency questionnaire. Odds ratios (ORs) and 95% confidence intervals (CIs) were estimated with multiple conditional logistic regression models, linking pattern scores and NPC risk. A high score of the PLS-DA derived pattern was characterized by high intakes of fruits, milk, fresh fish, vegetables, tea, and eggs ordered by loading values. We observed that one unit increase in the scores was associated with a significantly lower risk of NPC (OR_adj_ = 0.73, 95% CI = 0.60–0.88) after controlling for potential confounders. Similar results were observed among Epstein-Barr virus seropositive subjects. An NPC protective diet is indicated with more phytonutrient-rich plant foods (fruits, vegetables), milk, other protein-rich foods (in particular fresh fish and eggs), and tea. This information may be used to design potential dietary regimen for NPC prevention.

## Introduction

Nasopharyngeal carcinoma (NPC) is a rare disease in most parts of the world. Its age-adjusted incidence rate for both men and women is less than one case per 100,000 person-years [1]. However, NPC incidence in South-Eastern Asia is much higher [2]. Several cities in southern China, including Zhongshan, Guangzhou, and Hong Kong have very high incidence of NPC particularly in males (22, 27, and 21 per 100,000 person-years, respectively). Population in Taiwan demonstrates an intermediate risk of NPC (8.3 and 3.5 per 100,000 person-years for males and females, respectively) [3]. The incidence of NPC in Taiwan has decreased gradually over the past two decades [4], suggesting that environmental factors may play an important role in the etiology of this disease.

Similar to most types of cancer, multiple factors are involved in the etiology of NPC including Epstein-Barr virus (EBV) activation, genetic susceptibility and environmental factors [5]. Among them, EBV exhibits the strongest effect on NPC development. Previous studies have provided evidence that, among environmental factors, diet is associated with NPC [6]. For example, it has been suggested that Cantonese-style salted fish may be one of the causative environmental risk factors for NPC, whereas high intakes of fresh fruits and non-preserved vegetables are protective factors [6]. In addition, a few studies have investigated and found potential protective role of single nutrients on NPC [7–11] including vitamins A, C, E, carotenoids, and total dietary fiber. While the role of diet and nutrition have been hypothesized and studied, a comprehensive understanding is still lacking. A major challenge in conducting studies of single food or nutrient in relation to diseases is the high degree of correlation among dietary constituents [12]. Therefore, a dietary pattern approach has emerged to address the well-known issues of collinearity among various nutrients and interdependencies among foods and nutrients. To the best of our knowledge, there are only two studies which investigated the relationship between NPC and dietary patterns, identified through an exploratory factor analysis [13, 14]. Using factor analysis to extract information from food frequency data, Armstrong and colleagues [13] carried out a case–control study in a high-incidence area and found that NPC was positively associated with a dietary pattern rich in salted preserved food and pork/beef organ meat, but inversely associated with a vegetables/fruits-rich pattern. In a low-incidence area, Edefonti and colleagues [14] identified several nutrient-based dietary patterns and suggested that a pattern indicative of diets rich in animal products, sugar/candy, and fats and another pattern of starch-rich foods were positively associated with NPC risk, whereas the vitamin and fiber-rich pattern was inversely (although non-significantly) associated with NPC. However, these two studies did not take into consideration of the association between diet and outcome variable in the pattern-mining process. Certain relevant information may be missed. Therefore, we propose to used partial least-squares regression discriminant analysis (PLS-DA) as an alternative to principle component analysis (PCA)/factor analysis for deriving dietary patterns [15], since this method will find a linear combination of food frequencies that best separates the patients and controls, thereby allowing for the discovery of novel disease-related dietary pattern [16, 17]. Therefore, in the present study consisted of 319 matched case-control pairs, we tried to find a dietary pattern with the best discriminative power using PLS-DA method and examined the dose-response relationship between the dietary pattern scores and NPC.

## Materials and Methods

### Study subjects

Detailed information of the case-control study methods has been published elsewhere [18, 19]. Briefly, data were collected between July 1991 and December 1994 for individuals aged less than 75 years with incident, primary, histologically confirmed NPC and for age (within 5-years), gender, and residence area-matched controls with no history of NPC. In total, there are 378 cases and 372 controls identified. Of these, risk factor questionnaires were obtained from 375 (99%) cases and 327 (88%) controls. Most cases (>90%) were diagnosed as the WHO Types 2 or 3 (non-keratinizing and undifferentiated carcinomas), whereas the remaining cases were diagnosed as the WHO Type 1 (squamous cell carcinomas) [20]. The dietary analyses were limited to 319 incident NPC cases and 319 matched controls with complete information. Excluded were 56 cases and 8 controls with missing values. Baseline socio-demographic characteristics of individuals excluded and those remaining in the study were similar. Institutional Review Boards at the National Taiwan University in Taiwan and the National Cancer Institute in the United States (both under the NCI Special Studies IRB) approved the study protocol and informed consent. Written inform consent was obtained from every study participant.

### Data collection

Information on socio-demographic characteristics, smoking habit, alcohol consumption, residential history, and medical history were collected during face-to-face interviews by trained nurses using a structured questionnaire. For cases, interviews were conducted at the time of biopsy for histological confirmation of NPC and before treatment.

We used a 66-food-item quantitative food-frequency questionnaire (FFQ) to query about the dietary history for the time period 10 to 3 years before the diagnosis for cases or before the interview date for controls. For data analysis, these food items were grouped into 24 food groups (S1 Table), based on similar food group characteristics and nutrient contents [21–23]. Validation of a similar simplified FFQ designed for the Elderly Nutrient and Health Survey in Taiwan has been previously reported [24]. The major difference between this FFQ and the validated one is that this FFQ included additional questions concerning tea and preserved foods. Participants were asked to indicate their average intake frequency per day, week, month, or year or less than once per year. Using the Taiwan food composition database, total caloric intake was estimated by multiplying the intake frequency for each food item by the nutrient content of the food in an estimated usual portion size [23]. All the food frequency data of the above 24 food groups have been adjusted for total energy intake by the residual method [25] and standardized into a N (0, 1) distribution before being subjected to the dietary pattern analyses. The outliers located ≥5 standard deviations beyond the mean were excluded.

EBV status was defined based on the detection of various anti-EBV antibodies in serum samples including viral capsid antigens (VCA) IgA, EBV nuclear antigen 1 (EBNA1) IgA, early antigens (EA) IgA, DNA binding protein IgG, and anti-EBV DNase [11, 26–28]. Individuals positive for any one of the EBV biomarkers were classified as "seropositive".

### Statistical analysis

SAS software, version 9.4 (SAS Institute Inc.) was used for all statistical analyses. Demographic variables between cases and controls were compared using Student’s t-test for continuous variables and Pearson’s χ^2^ test for categorical variables. Conditional logistic regression was used to estimate odds ratios (ORs) and 95% confidence intervals (CIs).

For dietary pattern discovery, we used the PLS regression method. PLS is a method that works with two different sets of variables, called predictors and responses. This approach aims to identify the linear combinations of predictor variables Xi (that is, frequencies of the 24 food groups) which best discriminates the dependent variable, i.e., NPC status (disease vs no disease). Detailed description of the methods can be found from Hoffman et al. work [15]. The PLS regression method we used is the PLS-DA [17]. The PROC PLS statement in SAS was used to conduct PLS-DA analyses. The SAS code for the applied SAS procedure PLS is given in the S1 Text.

The dietary pattern identified was constructed further to a score by weighing each food frequency with factor loading values. Individual factor scores were then categorized into quartiles. For interpreting the dietary pattern, we emphasize those food groups with absolute values of loadings ≧0.20.

Finally, conditional logistic regression analysis was performed to determine whether the dietary pattern scores obtained by applying PLS-DA were significantly associated with the risk of being a NPC case. Three multivariable models were constructed with three sets of covariates (see the result section). The dietary pattern scores were treated both as continuous and as categorical variables, based on the quartiles in all study subjects.

In order to observe the characteristics of the 4 dietary groups (first to fourth quartiles of factor score, Q1-Q4), the age-adjusted mean values were obtained for certain potential confounders of NPC such as age, socio-demographics, lifestyle variables, and EBV infection status. Weekly consumption frequencies of food groups were additionally adjusted for total energy intake by the residual method [25].

Because greater than 95% of NPC cases were seropositive for EBV infection and because EBV is considered a necessary risk factor for the development of NPC, we repeat the above analysis after excluding those without EBV infection.

## Results

The distributions of the selected demographic characteristics of 319 NPC cases and 319 matched controls are shown in Table 1. The risk profile was similar to those reported in our previous studies [11, 18, 19, 29] based on similar subjects. The male proportion was around 69% for both cases and controls. There was no significant difference in cases and controls regarding age and gender, all of which were matched during recruitment. Cases tended to have more with ancestral origin of Fukienese and Hakka compared with that of the controls. The cases had lower education level compared with the controls. Smokers for more than 25 years had a significantly increased risk of NPC (OR_adj._ = 1.64; 95% CI = 1.01–2.68). A significantly increased risk was found to be conferred by a family history of NPC (yes vs. no, OR_adj._ = 6.96; 95% CI = 2.07–23.44). Exposures to formaldehyde, wood dust, and high intake of nitrosamines were associated with increased risks of NPC. Majority of cases (99.4%), but minority of controls were seropositive for anti-EBV markers compared to controls. No association was found between NPC risk and alcohol drinking habit.

**Table 1:** Sociodemographics, Lifestyle, and Viral Status of 319 Nasopharyngeal Carcinoma Cases and 319 Matched Controls.

Characteristic/Category	Cases	Controls	P-value ^a^	Adj. OR (95%CI)
	n (%)	n (%)		
Gender				
Male	220 (69.0)	220 (69.0)	1.00	
Female	99 (31.0)	99 (31.0)		
Age (mean ± SD), years	45.6 ± 11.6	46.0 ± 11.7	0.62	
Age, years				
<40	103 (32.3)	102 (32.0)	1.00	
40–49	93 (29.1)	94 (29.5)		
50–59	77 (24.1)	74 (23.2)		
60–69	42 (13.2)	45 (14.0)		
≧70	4 (1.3)	4 (1.3)		
Ethnicity				
Mainlander ^d^	34 (9.2)	68 (21.2)	<0.0001	1.00 (Ref.)
Fukienese	305 (82.2)	234 (72.9)		2.56 (1.57–4.18) ^b^
Hakka	32 (8.6)	19 (5.9)		3.13 (1.52–6.44) ^b^
Educational level				
Junior high school or below	189 (59.3)	160 (50.2)	0.02	1.00 (Ref.)
Senior high school or above	130 (40.7)	159 (49.8)		0.59 (0.41–0.86) ^c^
Smoking habit				
0–25 years	241 (75.6)	256 (80.3)	0.15	1.00 (Ref.)
Smoker ≧25 years	78 (24.4)	63 (19.7)		1.64 (1.01–2.68) ^c^
Alcohol drinking habit				
0–10 years	250 (78.4)	247 (77.7)	0.83	1.00 (Ref.)
≧10 years	69 (21.6)	71 (22.3)		0.99 (0.66–1.47) ^c^
Formaldehyde exposure				
No	260 (81.5)	278 (81.2)	0.05	1.00 (Ref.)
Yes	59 (18.5)	41 (12.8)		1.61 (1.02–2.53) ^c^
Wood dust exposure				
No	276 (86.5)	293 (91.9)	0.03	1.00 (Ref.)
Yes	43 (13.5)	26 (8.1)		1.83 (1.06–3.17) ^c^
Nitrosamine from foods ^e^				
Q1 (low)	64 (20.1)	80 (25.1)	0.18	1.00 (Ref.)
Q2	71 (22.3)	81 (25.4)		1.23 (0.76–1.99) ^c^
Q3	84 (26.3)	78 (24.4)		1.63 (0.98–2.70) ^c^
Q4 (high)	100 (31.3)	80 (25.1)		1.78 (1.11–2.88) ^c^
NPC family history				
No	296 (93.1)	313 (99.1)	0.0001	1.00 (Ref.)
Yes	22 (6.9)	3 (0.9)		6.96 (2.07–23.44) ^c^
Anti-EBV seropositive				
No	2 (0.6)	216 (68.8)	<0.0001	1.00 (Ref.)
Yes	313 (99.4)	98 (31.2)		243.60 (32.07->999) ^c^

Adj., adjusted; CI, confidence interval; OR, odds ratio; Ref., referent, SD, standard deviation.

^a^ Significant difference between cases and controls were assessed using a Chi-square test for categorical variables and Student’s t-test for continuous variables.

^b^ Adjusted for age.

^c^ Adjusted for age, and ethnicity.

^d^ Those who moved to Taiwan in the past few decades.

^e^ Nitrosamine index was calculated from food consumption. Index values were categorized into quartiles based on the distribution among the controls, and those in the lowest quartile were used as the referent group.

The factor loadings of the identified primary dietary pattern obtained from PLS-DA are shown in Table 2. Food groups with absolute values of loadings ≧0.20 were emphasized in the interpretation. We observed higher loading values from fruits, milk, fresh fish, vegetables, tea, and eggs in that order, each of which was inversely associated with the NPC; suggesting that these foods were less frequently consumed by NPC patients.

**Table 2:** Factor Loading Values Obtained by Partial Least Squares Discriminant Analysis Method, the Spearman Correlation Coefficients between Food Groups and Factor Scores.

Food Groups	Factor loadings ^a^,^b^	Correlation with Factor Scores
**Fruits**	**-0.43**	-0.43***
**Milk**	**-0.40**	-0.41***
**Fresh fish**	**-0.35**	-0.38***
**Vegetables**	**-0.32**	-0.27***
**Tea**	**-0.27**	-0.28***
**Eggs**	**-0.24**	-0.29***
Coffee	-0.19	-0.13*
Nuts	-0.18	-0.22***
Legumes	-0.16	-0.21***
Poultry	-0.15	-0.25***
Salted fish	-0.15	-0.11
Soybean products	-0.14	-0.22***
Seafood other small fish	-0.11	-0.23***
Dry bean products	-0.09	-0.08
Smoked foods	-0.09	-0.12*
Red meat	-0.08	-0.12*
Processed meat	-0.05	-0.06
Raw meat	-0.05	-0.18*
Processed vegetables	-0.05	-0.05
Sauces	-0.02	-0.14*
Animal oil for cooking	0.02	0.16*
Vegetable oil for cooking	0.06	0.04
Liver	0.13	0.03
Fried foods	0.19	0.04

* *p*-value < 0.05;

** *p*-value < 0.001;

*** *p*-value <0.0001.

^a^ Pattern was derived with partial least squares regression with nasopharyngeal carcinoma disease as the dependent variable and 24 food groups as the predictor variables. Food groups with absolute values of factor loadings ≧0.20 were marked in bold style.

^b^ The dietary pattern obtained from PLS-DA explained 4.8% of the variance in response variables, and 6.7% of the variance in all food groups.

We constructed a dietary pattern score by weighing food frequency with factor loadings values of all food groups. The distribution of potential confounders by quartiles of the dietary factor scores is shown in Table 3. Those in upper quartiles of factor scores were educated more. Amount of nitrosamine from foods and EBV seropositive proportion decreased across increasing quartiles. Individuals in the fourth quartile of the dietary pattern scores consumed on average 1–2 times of fruits, 5 times of vegetables, and 5 times of protein-rich foods per day, and 4 times of milk per week. Among the protein-rich foods, these people consumed 1 time of fresh fish and 1 time of eggs per day. In contrast, individuals in the first quartile consumed on average much less vegetables (4 times per day), and protein-rich foods (3 times per day); fruits (4 times per week), and milk (around twice per month). Among the protein rich foods, these people consumed much less fresh fish (3 times per week) and eggs (3 times per week).

**Table 3:** Distribution of Potential Confounders and Mean Food Frequency by Quartiles of Dietary Factor Scores.

	Quartiles of Factor Scores				
	Q1	Q2	Q3	Q4	
Variables	−11.1 (−13.5 to −2.1) ^a^	−16.0 (−18.1 to −13.6) ^a^	−21.2(−24.0 to −18.2) ^a^	−27.7 (−55.9 to −24.0) ^a^	P for Trend
Potential NPC confounder ^b^					
Age, years	46.2	45.4	46.0	45.9	1.00
Gender, male, %	67.8	64.0	67.9	78.7	0.67
Ethnicity, Fukienese, %	82.3	80.7	73.6	73.1	0.02
Senior high school or above, %	36.8	40.6	42.3	61.3	<0.0001
Smokers≧25 years, %	20.9	25.1	22.4	19.9	0.66
Alcohol drinker≧10 years, %	21.3	19.5	24.5	22.6	0.54
Formaldehyde exposure, %	16.9	13.9	18.8	13.1	0.61
Wood dust exposure, %	15.1	9.4	10.0	8.7	0.10
Nitrosamine ^c^, Q4, %	53.4	28.9	18.9	11.9	<0.0001
NPC family history, %	8.9	2.5	1.9	2.5	0.01
Anti-EBV seropositive, %	74.3	71.3	60.5	55.3	<0.0001
Food group (times/week) ^d^					
Fruits	4.0	6.0	8.0	10.0	<0.0001
Milk	0.6	1.0	2.3	4.0	<0.0001
Fresh fish	2.8	4.0	4.7	6.7	<0.0001
Vegetables	27.4	30.8	32.8	35.8	<0.0001
Tea	4.0	4.5	7.6	12.2	<0.0001
Eggs	3.3	3.7	4.7	5.7	<0.0001
Coffee	1.3	0.6	3.1	2.0	0.83
Nuts	1.7	1.9	2.6	3.6	<0.0001
Legumes	1.4	1.6	2.1	2.8	<0.0001
Poultry	1.1	1.5	1.9	2.4	<0.0001
Salted fish	0	0	0	0	0.91
Soybean products	4.8	5.0	6.1	7.6	<0.0001
Seafood other small fish	2.1	2.1	3.0	3.8	<0.0001
Dry bean products	0.7	0.9	1.1	1.1	0.08
Smoked foods	0.3	0.3	0.5	0.7	0.05
Red meat	7.8	6.8	8.6	8.9	0.06
Processed meat	0.3	0.3	0.5	0.4	1.00
Raw meat	0.1	0.1	0.2	0.4	0.04
Processed vegetables	0.7	1.1	1.2	1.1	0.55
Sauces	3.0	3.1	3.4	4.0	0.05
Animal oil for cooking	9.7	5.4	5.7	3.8	<0.0001
Vegetable oil for cooking	30.3	29.3	28.7	27.1	0.37
Liver	0.4	0.3	0.4	0.3	0.75
Fried foods	1.4	1.0	0.9	0.9	0.57

Q1, first quartile; Q2, second quartile; Q3, third quartile; Q4, fourth quartile.

^a^ Median and interquartile range of dietary factor scores.

^b^ Values are age-adjusted means or percent (%).

^c^ Nitrosamine index was calculated from food consumption. Index values were categorized into quartiles based on the distribution among the controls.

^d^ Values are age and total energy-adjusted means.

Table 4 presents the ORs and corresponding CIs for NPC by the calculated dietary pattern scores or by the quartiles. Three different multivariate models were constructed. We observed a significant and inverse association between the dietary pattern scores and risk of NPC after controlling for age (M1). This finding was observed when the dietary pattern scores were treated either as a categorical variable (quartiles) or as a continuous variable. One unit increase in dietary pattern scores was associated with approximately 30% lower risk of being NPC patient in the age-adjusted model (OR_adj._ = 0.70, 95% CI = 0.61–0.82 in M1). Similar results were found in M2 (adjusting for age, ethnicity, educational level, NPC family history, total calories, years of cigarette smoking, and exposures to formaldehyde and wood dust) and M3 (adjusting for covariates used in M2 and additionally for green tea and nitrosamine) [11, 29]. The association between the dietary pattern factor scores and NPC was only slightly attenuated in M2 (OR_adj._ = 0.71, 95% CI = 0.60–0.84) and M3 (OR_adj_ = 0.73, 95% CI = 0.60–0.88), respectively. The subjects whose diet conformed most closely to the dietary pattern (fourth quartile, Q4) had a NPC risk reduction by 62% compared with the subjects whose diet was most different from this pattern (first quartile, Q1) after controlling for all potential confounders (OR_adj._ = 0.38, 95% CI = 0.21–0.69 for Q4 versus Q1; *p* for trend 0.001 in M3).

**Table 4:** Odds Ratios (ORs) of Nasopharyngeal Carcinoma and Corresponding 95% CIs According to Quartiles of Factor Scores and Continuous Factors Scores from Partial Least Squares Discriminant Analysis.

	Quartile of Dietary Pattern Scores ^a^					Continuous
	Q1	Q2	Q3	Q4		
Subjects	Adj. OR (95% CI)	Adj. OR (95% CI)	Adj. OR (95% CI)	Adj. OR (95% CI)	P for Trend	Adj. OR (95% CI)
Cases / Controls	104/55	83/77	70/89	62/98		
Crude	1.00 (Ref.)	0.58 (0.37–0.94)	0.42 (0.27–0.67)	0.34 (0.21–0.54)	<0.0001	0.70 (0.60–0.81)
M1 ^b^	1.00 (Ref.)	0.60 (0.38–0.97)	0.44 (0.27–0.69)	0.35 (0.22–0.55)	<0.0001	0.70 (0.61–0.82)
M2 ^c^	1.00 (Ref.)	0.63 (0.38–1.06)	0.50 (0.30–0.83)	0.34 (0.20–0.59)	<0.0001	0.71 (0.60–0.84)
M3 ^d^	1.00 (Ref.)	0.67 (0.39–1.14)	0.51 (0.30–0.88)	0.38 (0.21–0.69)	0.001	0.73 (0.60–0.88)

Adj., adjusted; CI, confidence interval; OR, odds ratio; Q1, first quartile; Q2, second quartile; Q3, third quartile; Q4, fourth quartile; Ref., referent.

^a^ Median of factor scores among all subjects: Q1, −11.1; Q2, −16.0; Q3, −21.2; Q4, −27.7.

^b^ M1: adjusted for age.

^c^ M2: M1 with further adjustment for ethnicity, educational level, NPC family history, total caloric intake, years of cigarette smoking, and exposures to formaldehyde and wood dust.

^d^ M3: M2 with further adjustment for intake of green tea and nitrosamine.

## Discussion

The aim of this case-control study was to identify dietary pattern and to document the dose-response relationship between the identified primary dietary pattern scores and the risk of NPC. We identified a dietary pattern inversely associated with NPC risk, which is characterized by high consumption frequencies of fruits, milk, fresh fish, vegetables, tea, and eggs (in that order). The dietary pattern pinpoints the importance of the phytonutrient-rich plant foods (fruits, vegetables), milk, the protein-rich foods (in particular fresh fish and eggs), and drink (tea).

Previous studies investigating the effects of individual foods (or food groups) have consistently found that fresh fruits and leafy vegetables are protective factors against NPC in Chinese [13, 30–34], North Africans [35], and some low risk populations [8, 36], which were also confirmed by a meta-analysis study [37]. Many kinds of nutrients or food constituents rich in fruits and vegetables have been associated with reduced risk of cancer in general, including fiber, vitamins, folate, and carotenoid [6]. An inverse association between NPC risk and consumption of fresh fish and green tea was observed in our previous report [11]. The inverse relation between fish and cancer had been observed and described in reviews and meta-analyses [38, 39]. The potential mechanism of the protective effect of fish consumption maybe the well-known anti-inflammation effect of n-3 fatty acids [40]. In addition, tea consumption has been associated with a decreased occurrence of NPC in another case-control study conducted in southern China [41]. There is considerable evidence that extracts of tea and tea polyphenols have been shown to inhibit the formation and development of tumors at different organ sites in animal models [42]. However, apart from the above the above-mentioned protective associations (with vegetables, fruits, tea, and fish) and the previously reported risky association with Cantonese-style salted fish and other preserved foods in high incidence areas [6], the evidence on dietary components/dietary pattern and NPC is still limited.

Our study has derived a NPC protective dietary pattern characterized by two additional dietary items (milk and eggs) which were not previously reported. However, previous evidences on the effects of milk and eggs on NPC showed opposite results. The association between eggs and cancer mortality in general also showed inconsistent results [43] in epidemiological studies. The majority of the single-food-item studies reported null results [11, 32, 33]. Similar to our study, consumption of boiled or fried eggs in childhood was inversely associated with NPC risk in Algeria [44]. In contrast to our study, one study assessed the role of dietary patterns through a factor analysis and showed the animal product pattern, loading highly on milk, cheese, desserts, and eggs, was positively associated with NPC risk [14]. Likewise, Polesel et al. [36] reported a twofold increase in NPC risk in people within the highest quartile of consumption of eggs. Some cohort studies have reported positive associations of milk consumption with coronary heart disease [45] and prostate cancers [46].

The main concern when referring to adverse influence of milk consumption has been related to its saturated fat content. In addition, various exogenous hormones such as insulin-like growth factor 1 (IGF-1) are present in milk, which was suggested as a possible promotional effect for milk as a risk of hormone-dependent cancer, such as breast cancer in women and prostate cancer in men [47]. With the afore-mentioned results and conclusions, it is important to consider the U-shaped association between intake of milk and subsequent risk of cancers. The right-hand limb of the U is likely the well-known higher risk of cancers due to promotional effect of saturated fat and other hormone-like factors from excessive milk consumption. The left-hand limb of the U may indicate higher risk of cancers due to the lack of protective and nutritious effect from milk consumption. Milk consumption among Taiwanese has been much lower than that in western countries because of the cultural reasons and lactose intolerance. Therefore, subjects whose diet conformed with good pattern (with frequency of milk consumption around 1–4 times per week for those in the second to the fourth quartiles of factor scores) in the present study might be considered as light-to-moderate milk consumers by western standard. Our finding suggested that light-to-moderate milk consumption may protect people from NPC in region low in milk intake. With respect to egg consumption, Benn et al [48] used Mendelian randomization approach to show that very low plasma levels of low-density lipoprotein (LDL) cholesterol may be causally associated with an increased risk of cancer. Therefore, U-shaped association between the level of blood total or LDL-cholesterol and subsequent mortality has been suggested [49]. That is, higher levels of blood cholesterol have higher risk of death from coronary heart disease, but lower levels of blood cholesterol is also a risk marker for future cancer [50]. The inverse egg consumption association with NPC we found is consistent with these findings concerning cholesterol metabolism, although it is not clear whether it is the cholesterol or other food constituents in egg playing the central roles.

Given the strong evidence supporting a causing role of EBV in the development of NPC [5], EBV status may confound the observed association between dietary habits and NPC. We have assessed the association between dietary pattern and NPC among EBV seropositive individuals (313 cases and 98 controls). The association between NPC and dietary pattern scores remained statistically significant (data not shown). In other words, our study suggested that the composition of the dietary pattern we observed may play a protective role in the development of NPC even when people are infected with EBV.

Several research design limitations and methodology considerations should be noted. First of all, this was a retrospective study. Association does not equate directly to causality. Secondly, recall bias is a concern in case-control design. Therefore, the results should be further confirmed. Thirdly, FFQ provides direction of the association between diet and disease and yet it does not provide accurate estimates of the food frequency and food amount due to systematic errors associated with the FFQ methodology. Fourthly, since Chinese customarily share many dishes with family members; the portion size is usually small and the frequency is often high. Direct comparison of food consumption frequency with western studies may not be appropriate. Moreover, the staple was not addressed in this study since at the time white rice was the main staple which contributed over 92% among the staple foods [22]. Lastly, in contrast to PCA method which focuses soly on dietary pattern discovery by maximizing the variability explained for food frequency, PLS attempts to discriminant the diseased and the control as well as finding associated food patterns. The portion of people with the PLS-discovered pattern is usually smaller than those found by PCA.

In conclusion, this is one of the first studies on dietary pattern and NPC in Asian population, employing a novel approach, PLS-DA. We disclosed a protective dietary pattern with a nutrient-dense diet high in n-3 fatty acid and with balanced amount of good foods from both plant and animal origins. Since it is simply a Taiwanese diet with more phytonutrient-rich plant foods (fruits, vegetables) and more fresh fish and egg dishes accompanied by frequent tea drinking and morning milk, it can be easily adopted into the modern Taiwanese diet. Our findings suggests that this dietary pattern may be considered and trialed as a potential lifestyle regimen to prevent NPC.

## Supporting Information

S1 Table Food Items and Food Groups included in the Derivation of Dietary Patterns associated with Nasopharyngeal Carcinoma. ^a^ Fresh eggs, preserved eggs and salted eggs were grouped together for their similar cholesterol contents. ^b^ Fruits and 100% fruit juices were grouped together due to similar vitamin and mineral contents and the fact that less than 1% of the people even consumed 100% fruit juices at the time when the study was carried out.
(DOCX)

S1 Text SAS Code for Deriving Dietary Patterns. (DOCX)

## Abbreviations

aOR: adjusted odds ratio
CI: confidence interval
NPC: nasopharyngeal carcinoma
PLS-DA: partial least square discriminant analysis
