# Supplementary material for: Partial Least Square Discriminant Analysis Discovered a Dietary Pattern Inversely Associated with Nasopharyngeal Carcinoma Risk
Source: PLoS One. 2016 Jun 1;11(6):e0155892. doi: 10.1371/journal.pone.0155892 (PMC4889039; doi:10.1371/journal.pone.0155892)
Supplement: S1 Table — a Fresh eggs, preserved eggs and salted eggs were grouped together for their similar cholesterol contents. b Fruits and 100% fruit juices were grouped together due to similar vitamin and mineral contents and the fact that less than 1% of the people even consumed 100% fruit juices at the time when the study was carried out. (DOCX) [file pone.0155892.s001.docx]

S1 Table. Food Items and Food Groups Included in the Derivation of Dietary Patterns Associated with Nasopharyngeal Carcinoma.

| Food Groups | Food Items | |
| --- | --- | --- |
| Processed meat | | Ham, sausages, hot dogs |
| Smoked foods | | Smoked foods, barbecue, bacon |
| Raw meat | | Sliced raw fish, sliced raw meat |
| Red meat | | Pork and beef |
| Liver | | Polk liver, chicken liver, duck liver |
| Poultry | | Chicken, duck and goose |
| Fresh fish | | All fresh fish and eels |
| Seafood other small fish | | Small fish with edible bones, oyster, mussel, clam, shrimp, huazhi, squid, crab, cucumber |
| Salted fish | | Salted fish |
| Eggs ^a^ | | Fresh eggs, preserved eggs and salted eggs |
| Milk | | Milk |
| Tea | | Flower tea, green tea, black tea and oolong tea |
| Coffee | | Coffee |
| Fruits ^b^ | | All fresh fruits, 100% food juices |
| Vegetables | | Leafy vegetables, dark green vegetables, carrots, yellow squash, yams |
| Processed vegetables | | Salted vegetables, preserved vegetables, pickled vegetables |
| Legumes | | Pea pods, sweet pea pods, green beans, string beans, fresh beans |
| Nuts | | Peanuts, peanut products, melon seeds, almond, walnuts, cashews |
| Dry bean products | | Red bean products, green bean products, black bean products |
| Soybean products | | Soy beans, tofu, bean curd products, soybean milk, preserved bean curd, fermented bean curd |
| Sauces | | Spicy chili sauce, barbecue sauce, sweet chili sauce, fermented soybean paste, miso |
| Animal oil for cooking | | Lard etc. |
| Vegetable oil for cooking | | Soybean oil, sesame oil, camellia, olive oil, etc. |
| Fried foods | | Fried foods |

^a^ Fresh eggs, preserved eggs and salted eggs were grouped together for their similar cholesterol contents.

^b^ Fruits and 100% fruit juices were grouped together due to similar vitamin and mineral contents and the fact that less than 1% of the people even consumed 100% fruit juices at the time when the study was carried out.
