# Supplementary material for: Partial Least Square Discriminant Analysis Discovered a Dietary Pattern Inversely Associated with Nasopharyngeal Carcinoma Risk
Source: PLoS One. 2016 Jun 1;11(6):e0155892. doi: 10.1371/journal.pone.0155892 (PMC4889039; doi:10.1371/journal.pone.0155892)
Supplement: S1 Text — (DOCX) [file pone.0155892.s002.docx]

S1 Text. SAS Code for Deriving Dietary Patterns.

Our data file named ‘diet’ contains 24 food groups denoted by FGP1, …, FGP24 and the disease status denoted by NPC. The following SAS code was used to construct dietary patterns by using the PLS method:

/*Transform category values into dummy variables*/

data p_ex;

set diet;

if disease = ’1’ then do; y_a = 1; y_b = 0; end;

if disease = ’0’ then do; y_a = 0; y_b = 1; end;

run;

/*Standardize food variables with zero mean and unit standard deviation */

proc standard data = p_ex mean = 0 std = 1 replace

print out = zscore;

run;

/*You can set cv = split for determining the appropriate number of principal components*/

proc pls data = zscore method = pls missing = none details;

model y_a y_b = FGP1-FGP24/solution;

output out = pattern xscore = scorex yscore = scorey;

ods output ModelInfo = MI_cv;

ods output PercentVariation = PV_cv;

ods output CenScaleParms = CSP_cv;

ods output XLoadings = XL;

ods output XWeights = XW;

run;
